# Does ethics really matter to the sustainability of new ventures? The relationship between entrepreneurial ethics, firm visibility and entrepreneurial performance

**DOI:** 10.1371/journal.pone.0226920

**Published:** 2020-01-28

**Authors:** Li Ma, Yue Cao, Dake Jiang, Yang Gao, Xiaomin Du

**Affiliations:** 1 School of Business, Dalian University of Technology, Panjin, China; 2 Department of Economic Management, Yingkou Institute of Technology, Yingkou, China; Shandong University of Science and Technology, CHINA

## Abstract

This paper aims to explore whether entrepreneurial ethics can improve entrepreneurial performance in new ventures. The dynamic impact of entrepreneurial ethics on entrepreneurial performance (survival and sustainable growth) is investigated from an institutional perspective, and the moderating role of firm visibility between them is explored. From different regions of China, 296 valid questionnaires are obtained and analyzed. We find that entrepreneurial ethics is adverse to the survival of new ventures but conducive to their sustainable growth of new ventures. We also find that high firm visibility can help entrepreneurial ethics be more effective in improving entrepreneurial performance. This study provides a new insight to explain the theoretical controversy of entrepreneurial ethics and provides guidance for the establishment of the internal ethical structures of new ventures. Suggestions for government and industry regulators on the management of entrepreneurial ethics are also provided.

## Introduction

It is generally agreed among researchers that business ethics can promote performances of mature enterprises [1]. For external stakeholders, business ethics can help enterprises establish a good reputation, increase the brand recognition of consumers, expand market share, and obtain the approval of investors and suppliers [2–3]. For internal stakeholders, business ethics can improve employee job satisfaction and organizational commitment and enhance team performance [4].

However, the above conclusions are drawn mostly from the study on mature enterprises, rarely on new ventures; actually, whether ethics is conducive to improving the performance of new ventures is still controversial [5]. Some studies suggest that for new ventures burdened with the liability of newness and a certain cost paid, it is difficult to obtain the due benefits through business ethics and corporate social responsibility (CSR) practice; thus, their survival and improvement of performance cannot be ensured [6–8]. Other studies suggest that entrepreneurial ethics can improve the financial and non-financial performance of new enterprises, thus helping new enterprises earn entrepreneurial success [5,9]. It can be seen that the relationship between entrepreneurial ethics and entrepreneurial performance is still unclear. In addition, in the research paradigm of ethics and performance, there is a lack of research relating institutional factors as situational variables [10–11]. In fact, studies [12–13] have shown that the concept of ethics comes from institutional theory and is a standard to guide the behavior of enterprises after the integration of formal and informal institutions within enterprises. Therefore, exploring the relationship between ethics and new ventures’ performance by investigating beneficial factors of the institution on it can further exert ethics’ advantage on the ventures.

Thus, this paper aims to explore two questions: this paper aims to investigate: (1) whether entrepreneurial ethics is beneficial to the improvement of entrepreneurial performance; (2) how the key factors at the micro-level of the institution affect the relationship between entrepreneurial ethics and entrepreneurial performance based on from the institutional perspective, it explores. Answering these two questions can make up for the research gap of new ventures in the field of business ethics [14] and provide new insights for the success of entrepreneurship by maximizing the benefits of ethics. To answer the first question, firstly, entrepreneurial ethics is measured from the perfection of the internal ethical structure of enterprises instead of ethical practice. It is reasonable because the ethical structure is the foundation of the formation of ethical values [12–13]; such measurement can better reflect the source and essence of entrepreneurial ethics [13]. Secondly, this paper divides entrepreneurial performance into two dimensions: survival and sustainable growth; the impact of entrepreneurial ethics on these two entrepreneurial performances is compared to more comprehensively examine its dynamic impact on entrepreneurial performance. To answer the second question, this paper introduces the important variable of institutional factors, firm visibility, because firm visibility is the premise of stakeholders’ response to corporate behavior, and is the key factor in helping enterprises obtain legitimacy and then form the institutional environment for ethics [15]. This paper examines the moderating role of firm visibility in the relationship between entrepreneurial ethics and entrepreneurial performance, making up for the research of institutional perspective in the relationship between ethics and performance [10–11]. A sample of 296 Chinese new ventures was investigated in this paper.

In sum, the conclusion of this study makes several contributions to the literature. First of all, this paper provides a new perspective on the measurement of entrepreneurial ethics. Previous studies have mainly used the ethical practice to measure the moral stance of new ventures, ignoring the fact that ethical structure forms the basis for the ethical practice and climate of new ventures [13]. This paper enriches the perspective of entrepreneurial ethics research and contributes to the theoretical development of entrepreneurial ethics. Secondly, this paper finds that entrepreneurial ethics has a “two sides” effect on entrepreneurial performance, providing a dynamic way to handle the contradictory effect of entrepreneurial ethics on enterprises. Different from the previous studies only examining the static impact of entrepreneurial ethics on financial and non-financial performance, the dynamic way breaks through the single cognition of being “good” and “bad” of entrepreneurial ethics. Finally, this paper finds the important role of institutional factors in the relationship between entrepreneurial ethics and entrepreneurial performance, filling the research gap of institutional perspective in the ethics-performance research paradigm. This paper also finds that firm visibility has a positive regulatory role in the relationship between entrepreneurial ethics and entrepreneurial performance. From the institutional level, we find more factors contributing to ethics’ benefits and explain the different conclusions of the entrepreneurial ethics research under different institutional backgrounds.

## Theoretical background and hypothesis

### Entrepreneurial ethics

Entrepreneurial ethics constitutes a reasonable and acceptable norm for entrepreneurs [16]. Since the 1990s, researchers on entrepreneurship have begun to realize the significance of entrepreneurial ethics for the sustainable development of the global economy, and the research on entrepreneurial ethics has gradually risen. At present, research on entrepreneurial ethics mainly investigates entrepreneurs at the micro-level and new ventures at the macro-level [17]. For entrepreneurs, it is found that they tend to pursue economic interests and ignore ethics [18] due to the special ethical dilemma posed by pressure on resources [19] and pursuit of success and happiness [20].

However, there is relatively little research on new ventures; most of them focus on the impact of entrepreneurial ethics on organizational performance [17]. On this issue, previous studies have been controversial. Some Researchers suggest that ethical practice is “expensive” for new ventures, the liability of newness may weaken some positive effects and intensify some negative effects of CSR activities on new ventures’ financial performance [21]. They also suggest that there is often little penalty for “bad” behavior in an entrepreneurial firm [22]. Ethical practice in new ventures is not instrumental in promoting sales [23], and the capital investment of CSR practice has an adverse effect on the financial performance of new ventures [7]. In contrast, some studies show that entrepreneurial ethics can help enterprises win the trust of customers, and retain them in the long term [24], ultimately improving entrepreneurial performance [5, 9, 25]. However, most current research investigates ethical practices and ethical decision-making [5, 9, 23], while few studies have investigated the impact of the perfection of ethical structure on the performance of new ventures. The ethical structure is the basis for creating an ethical environment and regulating the behavior of employees and entrepreneurs. However, we should ask whether it is necessary for enterprises to establish a normative ethical structure at the start-up stage and whether the establishment of an ethical structure is conducive to the improvement of entrepreneurial performance. Relevant research on these points is scarce.

**Fig 1:**
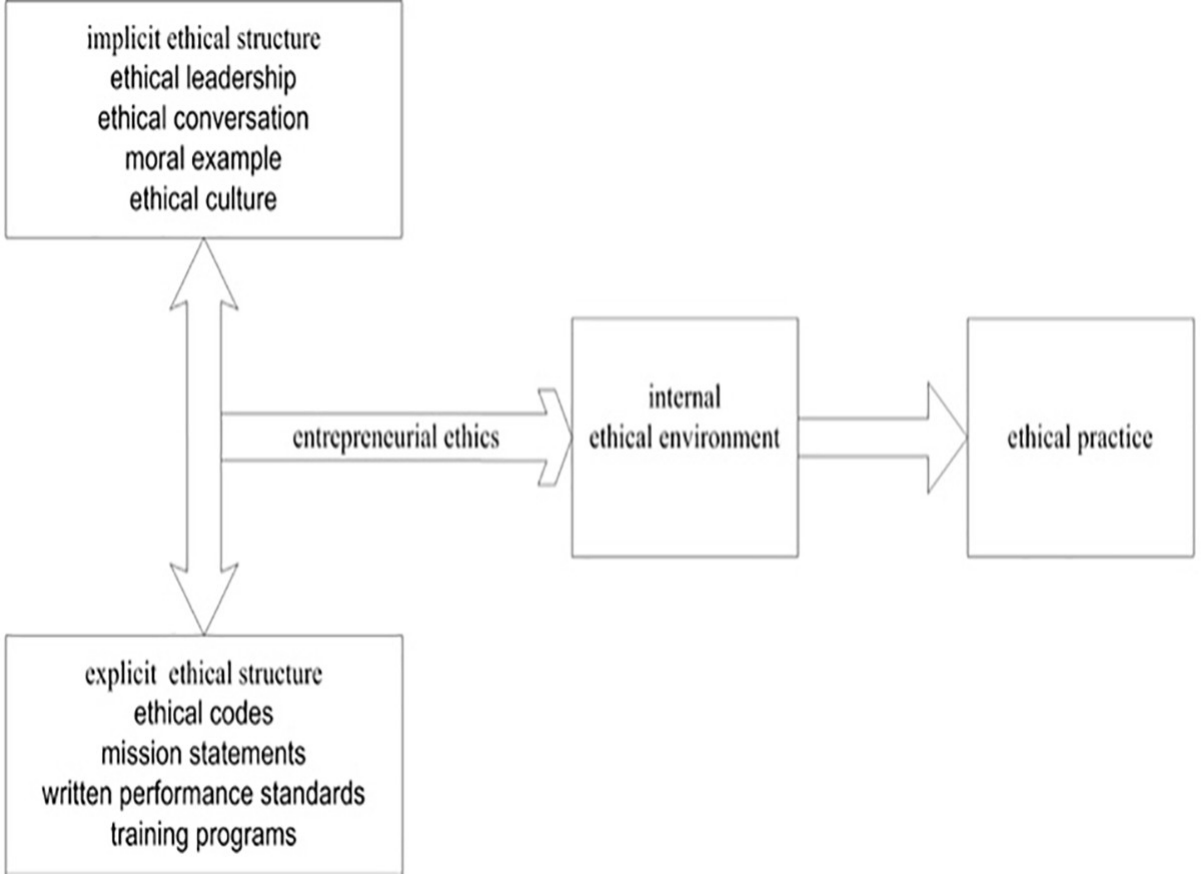
The impacts of ethical structures on ethical practice.

The previous study shows ethical structures can be classified as two kinds: implicit (informal) structure and explicit (formal) structure in new ventures [13]. The explicit ethical structure is clear to outsiders and has been called a compliance program, which means that ethical behavior is clearly expressed [26] and includes an ethical code, ethical training, and an ethical mission. The implicit ethical structure means that ethical behavior is implied, not directly expressed [26], and is largely invisible to outsiders. This is composed of ethical leadership, ethical conversation, moral example, and so on. The main difference between explicit and implicit ethics lies in the form of expression. Explicit ethics are more obvious, direct, clear and specific, and it is in the form of a formal system and norm [27]. Implicit ethics is vaguer, indirect, and permeated in daily work, and it is an informal system [28].Although existing studies have revealed that both ethical structures could have an influence on the organizational commitment and team benefit of employees, the independent study on the single ethical structure ignores the interaction effect of the two ethical structures [29]. Their multi-dimensional interaction reflects their ethical culture [28], which is the source of the ethical climate of enterprises and the embodiment of the values of new ventures [13]. Therefore, this paper defines entrepreneurial ethics as an ethical value of entrepreneurial enterprises from the perspective of the internal ethical structure of enterprises. The ethical value of enterprises is the embodiment of entrepreneurial ethics at an organizational level, and the concentrated expression of this value then constitutes the ethical environment generated by the multi-dimensional interaction of the ethical structure in a new venture. The ethical environment forms the basis for ethical decision-making and ethical practice. Fig 1 illustrates the elements of the two ethical frameworks and their impacts on decision-making related to ethical practice in enterprises.

### Entrepreneurial performance

Entrepreneurial performance can reflect the achievements and output of entrepreneurs in running enterprises [30, 31], and it is the only criterion to test whether the entrepreneurial activity of entrepreneurs is growing. The evaluation indexes of entrepreneurial performance can be divided into static and dynamic aspects. The static perspective refers to the evaluation of performance from the perspective of the static section, which is often divided into financial indicators and non-financial indicators [32]. Common indicators include the development of new products or services, attracting and retaining talents, customer satisfaction, employee relations, sales growth rate, and market share [33]. Research that is based on the dynamic perspective not only covers profitability but also focuses on the growth of new ventures [34], which in turn shows that enterprises sacrifice short-term profit for long-term growth.

However, current studies about entrepreneurial ethics mostly measure entrepreneurial performance from a static perspective, ignoring the dependence of business ethics and CSR practice on time accumulation and failing to fully reflect the impact of entrepreneurial ethics on the entrepreneurial development process. Regarding entrepreneurial performance, Chrisman et al. [35] showed that there are two dimensions of it: survival and success. Covin and Slevin [34] divided it into profitability and growth. This paper draws lessons from previous studies; we measured entrepreneurial performance in terms of survival and sustainable growth. Survival is the absolute measure of corporate performance and reflects the current operating status and is a relatively short-term concept. A company below a certain minimum level of performance is going bankrupt [36]. Sustainable growth is the core factor of the success of small- and medium-sized enterprises (SMEs) [37] and affects future business development, management activities and competitive advantages; it is a relatively long-term concept [38]. Therefore, dividing entrepreneurial performance into survival and sustainable growth not only reflects the development of the entrepreneurial process but also shows the dynamic impact of entrepreneurial ethics on entrepreneurial activities.

### Firm visibility

Firm visibility comes from institutional theory [39–40], and it is the premise for stakeholders to respond to corporate behavior [15]. As a unique corporate attribute, it reflects the status, exposure, and attractiveness of an enterprise [41–42]. There are three main reasons why this paper includes firm visibility into the research framework. Firstly, business ethics, as an enterprise strategy, its results will be affected by the external environment [10, 43]. Previous studies mainly focused on the industry environment and market environment of the enterprise, few on the environment from an institutional perspective [11]. However, it is more meaningful to explore the regulating role of institutional factors on the relationship between ethics and performance. Because the concept of ethics is derived from the internal ethical institution, forming the enterprise behavior standard after the integration of formal and informal institutions [12–13]. Therefore, ethics is logically the same as the external institutional environment [43], which belongs to the institutional level. Bringing institutional perspective into the research framework and exploring beneficial factors in institutional environment can enhance effectiveness of ethics in the performance of organization.

Secondly, the institutional theory holds that the essence of new venture growth is to overcome the “legitimacy threshold”, obtain resources, and constantly embed into the institutional environment [44]; obtaining legitimacy is important in the process of obtaining stakeholder recognition [45]. However, only when the enterprise is visible, the stakeholders would give feedback and recognition to the behavior of the firm [46], and then new ventures obtain legitimacy. Therefore, firm visibility is the premise for the legitimacy and institutional recognition of new ventures. Furthermore, firm visibility is the bridge between the internal behavior of new ventures and the external institution and plays a key role in the transformation of entrepreneurial ethics into entrepreneurial performance.

Thirdly, previous studies have confirmed the positive effect of firm visibility. The higher the firm visibility is, the more interaction between the company and stakeholders could increase [15]. This high interaction could enhance stakeholders’ attention and understanding of the enterprise and its operational activities; thus, meaningful feedback can be produced [47–48]. At the same time, with higher firm visibility, more benefits would be obtained from CSR practice [49]. Such benefits could be growing digital and representation effects [46], enhancing stakeholder recognition, improving financial performance [15] and gaining more investment for research and development expenditure [50], and promoting more enterprise innovation [51]. Therefore, we can infer that firm visibility is the key institution factor that affects the impact of ethics on performance. Bringing firm visibility into the research framework can help us to explore more benefits of ethics from the perspective of the institution and provide guidance for the coordination of external environment and internal policies for enterprises.

### Entrepreneurial ethics and entrepreneurial performance

#### Entrepreneurial ethics and survival of new ventures

(1) Implicit ethical structure and survival of new ventures

Implicit ethical structure such as ethical leadership can improve financial performance by increasing employee commitment and external stakeholder recognition [52], but it is difficult for an implicit ethical structure to have a positive impact on entrepreneurial performance in the short term. First of all, new ventures have the disadvantage of new entry and low influence, which means that it is difficult for them to release positive signals to the outside world through CSR practice and obtain benefits [21]. Secondly, from an internal perspective, the effect of ethical leadership which is one element of the implicit ethical structure is time-delayed [53] and has no direct influence on short-term financial performance [54]. On the other hand, moral leaders and ethical culture are unconducive to the short-term survival of new ventures. Ethical entrepreneurs operated with higher ethical standards may forgo short-term market expansion. Ethical leadership that implements a sustainable business strategy for stakeholders may sacrifice short-term interests [55]. Employees who work in an ethical culture have higher ethical standards and make choices according to a set of rules. However, employees who work for companies in which ethics play only a small part, or no part at all, actually sell more by misrepresenting the product. The following hypothesis is therefore put forward:

**H1.** An implicit ethical structure has a negative impact on the survival of new ventures.

(2) Explicit ethical structure and survival of new ventures

Downe et al. [55] pointed that one of the purposes of developing an explicit ethical structure is to regulate the ethical behavior of employees; however, some study has found that formally codified ethics may not be appropriate to guide behavior in new and unpredictable circumstances [56]. The role of ethical codes needs to be combined with an implicit ethical structure, such as ethical leadership, ethical dialogue and so on [57]. Leaders should set an ethical example for employees, and corporate culture needs to benefit from ethical codes of conduct [58]. It is thus a relatively lengthy process for explicit ethical structure to come into play. In addition, it takes a lot of time and money for an organization to develop these structures in the early stage of setting up a business [56]. A long-time is also needed to obtain returns, while the expenditure is both considerable and the process is time-consuming. Bureaucratic control reduces the flexibility and responsiveness of small companies [56], so that explicit ethical structure has no significant positive influence on the guidance of employees’ ethical behaviors in a new venture in the short term, and requires huge expenditure at the early stage. This is unconducive to the improvement of organizational flexibility and responsiveness. The following hypothesis is therefore put forward:

**H2.** An explicit ethical structure has a negative impact on the survival of new ventures.

(3) Interaction of the two ethical structures and survival of new ventures

The two ethical structures–implicit and explicit–together constitute the ethical environment of new ventures. They promote each other to produce a marked effect and strengthen a just ethical environment. However, the development of the ethical structure may be detrimental to the short-term survival of new ventures. First of all, it is difficult for entrepreneurial ethics to make a difference in terms of profit in the short term. Market forces do not price socially responsible characteristics in the short run [59]. It is, moreover, difficult for new ventures to obtain a good reputation through ethical practice, and the efforts to establish an upright corporate citizenship image often fail [60]. Secondly, the development of an ethical structure increases operating costs for enterprises. Also, new ventures may escape public scrutiny, and the amount of money the firms make through ethical misconduct far outweighs the penalty awarded to them [61]. Therefore, new ventures need enormous financial and human resources to build an ethical structure and form a sound moral working environment, neither of which brings due benefits in the short term. On the contrary, irresponsible enterprises may gain more sales and profits because of their speculative activities. The following hypothesis is therefore put forward:

**H3.** Entrepreneurial ethics have a negative impact on the survival of new ventures.

#### Entrepreneurial ethics and sustainable growth of new ventures

(1) Implicit ethical structure and sustainable growth of new ventures

In order to promote sustainable growth, new ventures need to gain legitimacy and committed followers, and it takes time to change the ethical climate in new ventures [62]. In the long term, implicit ethical structures can increase the organizational commitment of employees, attract and retain talents, and promote employee innovation, as well as being conducive to the sustainable growth of new ventures. Firstly, ethical leadership and ethical culture enable employees to perceive fairness and improve their job satisfaction, organizational commitment, and trust [63]; it can also reduce the turnover rate of employees [64]. Secondly, they help new ventures to attract high-quality talents. New ventures that have been established for a short time lack a good reputation and a track record. The higher the moral level of the entrepreneur, the more potential new members may think that the working climate within this enterprise is both fair and honest, thus improving their value judgment of the new venture and increasing their willingness to join the enterprise. Thirdly, an implicit ethical structure such as ethical leadership and ethical culture can promote innovation on the part of the employees. Entrepreneurs with high ethical standards create an ethical climate that caters to the interests and well-being of others, thus increasing employees’ spirit of adventure and sense of psychological security, and promoting employee innovation [65–66].

**H4.** An implicit ethical structure has a positive impact on the sustainable growth of new ventures.

(2) Explicit ethical structure and sustainable growth of new ventures

First of all, an explicit ethical structure that involves a bona fide ethical code would reduce the unethical behavior of employees in the long term. The effect of ethical training on reducing such behaviors is gradually enhanced over time [67], and this is of great significance for the sustainable growth and stability of an enterprise. Secondly, an explicit ethical structure involves an ethical statement, such as publicizing the ethical ideas of enterprises. It helps present a moral corporate image to the outside world and helps new ventures to obtain a good reputation [68]. New ventures can benefit from a good reputation and increasing trust from business partners, customers, and other stakeholders, by which new ventures could not only increase sales through consumer trust in the business and its products but also establish a long-term connection with business partners [69]. It can be concluded that companies with an explicit ethical structure can, therefore, gain more competitive sustainable advantages during the growth process after overcoming the pressures of early-stage investment over a certain period of time.

**H5.** An explicit ethical structure has a positive impact on the sustainable growth of new ventures.

(3) Interaction of the two ethical structures and sustainable growth of new ventures

When new ventures overcome early pressures in terms of the cost involved in building an ethical structure, the two structures promote one another in the long term and work together to promote venture’s growth. First of all, an implicit ethical structure can play a better role if there is an explicit ethical structure in place. Ethical ideas embodied in the implicit ethical structure are not directly expressed, so a venture needs an explicit ethical structure to formally express its moral intentions both to internal staff and external stakeholders alike. On the one hand, an explicit ethical structure can regulate the ethical behaviors of entrepreneurs and employees and reduce any immoral decisions. On the other hand, the practice of CSR without communication with external stakeholders would not have a positive impact on performance [70]. Through media advertising, an explicit ethical structure such as an ethical statement or ethical mission can be publicized to the outside world, thus can establish an ethical corporate image and gain legitimacy [69]. Secondly, an explicit ethical structure is improved by an implicit ethical structure. If explicit ethics and informal signals from a weak ethical culture are not consistent [71], then the mere existence of superficial ethical codes in new ventures can lead to low corporate performance [69]. Once external stakeholders find that an enterprise’s words are not matched by deeds, the loss of reputation can be extremely costly. Therefore, the two ethical structures support one another, which is conducive to the long-term stability and the sustainable development of ethical enterprises. The following hypothesis is therefore put forward:

**H6.** Entrepreneurial ethics have a positive impact on the sustainable growth of new ventures.

#### The moderating role of firm visibility

Firm visibility can weaken the negative impacts of entrepreneurial ethics on the survival of new ventures. It is also conducive to increasing the interaction between enterprises and stakeholders. New ventures with high firm visibility tend to have ethical practices that are more likely to be known to the outside world. It can, therefore, create a positive reaction on the part of consumers and investors to ethical behaviors. For example, it can increase consumers’ understanding of a company’s products and their payment premium [48], restrict opportunistic behavior, and obtain positive reviews from partners and investors [47]. Therefore, new ventures with high visibility can obtain more positive feedbacks from stakeholders as a result of ethical behavior, and obtain sales profits and investment needed for survival, which make up for the investment in establishing an ethical structure in the early stage. The following hypothesis is thus put forward:

**H7.** Firm visibility can weaken the negative impact of entrepreneurial ethics on the survival of new ventures.

In addition, entrepreneurial ethics play a more significant role in improving sustainable growth in new ventures with high visibility. In the long term, enterprise ethics can gain a good reputation for a firm, increase customer loyalty and obtain the trust of partners. High firm visibility increases the attention of potential consumers and employees and attracts potential alliance partners [47]. At the same time, due to an enhanced reputation, more well-known companies are likely to get more profit [46]. Visible social responsibility can improve the ability of enterprises to maintain a competitive advantage, achieve a strategic dialogue with stakeholders and form a good reputation [72]. Therefore, high firm visibility increases the ability of new ventures to gain a better reputation, which is conducive to the long-term growth and development of enterprises.

**H8.** Firm visibility can strengthen the positive influence of entrepreneurial ethics on the sustainable growth of new ventures.

**Fig 2:**
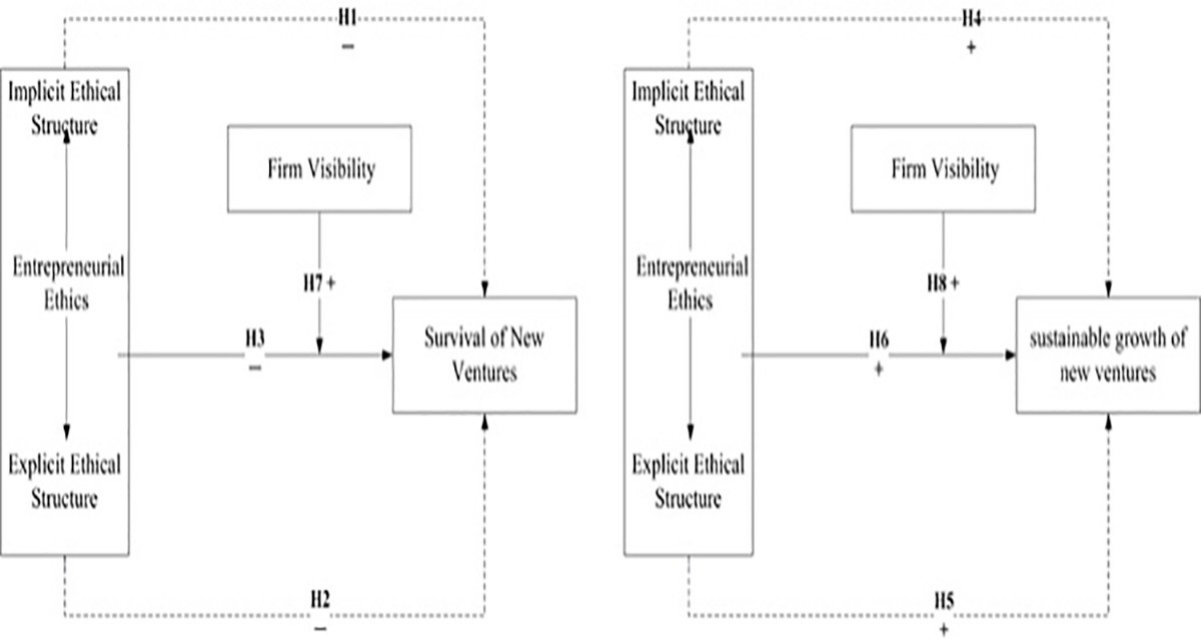
The overall research framework.

Fig 2 shows the overall research framework.

## Methodology

### Measures

Each scale for the variables was based on previous scales or definitions in the literature on the subject. All items were measured on a Likert five scale (from 1 = “strongly disagree” to 5 = “strongly agree”; from 1 = a large decrease to 5 = a large increase).

The measurement of the development of ethical structure was based on the research conducted by Morris [13]. We carried out item deletion and semantic modification according to the situation in China, including five items about implicit ethical structure: (1) In our firm, the top manager is concerned about ethics; (2) Candid ethical dialogue takes place in our firm between management and employees; (3) In our firm, ethics is a topic of conversation among employees; (4) There are formal rewards for exemplary ethical behavior in our firm; (5) Stories concerning ethical employees are broadcasting throughout our firm. Explicit ethical structure is, in turn, represented by four items: (1) There are ethical mission statements in our firm; (2) There are ethical codes of conduct in our firm; (3) There is a manager responsible for ethical issues in our firm; (4) There are training programs on ethical behavior in our firm.

In this paper, the entrepreneurial performance was measured from two aspects: survival and sustainable growth. The measurement of the survival of new ventures was based on Naidoo’s scale [73], and included five items: (1) Companies can cope with industry crises; (2) Companies can withstand cyclical changes to the economic environment; (3) A company’s market segments can respond to changes in industry development; (4) Companies can quickly recover from the shock of market changes to previous levels. The sustainable growth of new ventures was measured according to five items which referred to Sharu and Chandler’s research [74–75]: (1) The market share of the company’s products is growing rapidly; (2) The company’s sales are growing rapidly; (3) The company’s profits are growing rapidly; (4) The number of employees is growing rapidly.

Based on Burke’s definition [76], this paper measured firm visibility according to the degree of concern shown by stakeholders. Therefore, according to Murillo-Luna et al. [77], the scale was adapted to classify the types of stakeholder, adding “attention (visibility)” into each category, and measuring each category with one item, thus forming a dimension composed of five items as follows: (1) Investors and shareholders pay close attention to our firm; (2) Labor Unions pay close attention to our firm; (3) External stakeholders such as consumers and suppliers pay close attention to our firm; (4) Regulatory agencies pay close attention to our firm; (5) External organizations such as the media and the community pay close attention to our firm.

In order to avoid the model being influenced by external factors, three control variables were chosen: size, year, and industry. Details of the survey questions are in S1 File.

### Sample and data collection

In the sample selection, potential samples were identified from the National Small and Medium-sized Enterprise Service Platform. The platform is managed by the Ministry of Industry and Information Technology of the People’s Republic of China, and there are a large number of directories and basic information of small and medium-sized enterprises (SMEs) in this platform. According to Li and Atuahene-Gima [78], we choose the enterprises under eight years of the platform as the new ventures. To ensure that our sample represents a larger population, multiple locations with different levels of economic development (including southern, northern, coastal and inland regions of China) were selected. Thus, the influence of regional economic development on the study could be avoided. As for the sample size, Comrey and Lee stated that a sample size of 200 is reasonable, 300 is good [79]. Meanwhile, due to the particularity of network survey, the sample size is also often increased by 30% to compensate for no response [80]. Therefore, the potential sample in this study was set as 400.

The data were gathered in three phases. In the first phase, the enterprises were divided into four categories according to the regions in the platform: South Coast, North Coast, South inland and North inland. Then, 100 enterprises under eight years (a total of 400 enterprises) were selected respectively from each region by using simple random sampling technology, which meets the random standard. Their contact information was obtained through Tianyancha, a professional enterprise credit query platform in China. The second step was to contact the sample company by phone, email, and so forth. Senior managers of the company are invited to participate in the survey because they are trusted to have more information about the companies and play a critical role in their management. In the survey process, we indicated the purpose of the research and stated that our research was sponsored by the National Social Science Foundation, guaranteeing that the survey data would be kept confidential and only be used for academic research. Finally, their Wechat (an online chat App in China) accounts and email addresses were obtained so that official questionnaires could be distributed through the network.

The third step was to issue the formal questionnaire. A questionnaire link through the questionnaire collection tool was formed and subsequently sent to the senior manager through email and Wechat. The advantage of this collection method is that we are able to see the response of the questionnaire in the background, including the collection number of the collected questionnaire, filling time and other related information. In the whole process, most managers were given sufficient instructions and guidance about the scale items. It took us 11 months from the beginning of target enterprise screening and information collection to the end of questionnaire collection, from February 2018 to January 2019, making a total of 332 questionnaires. In order to ensure the high quality and validity of the questionnaires, 36 were removed for various reasons (interruptions during filling, short filling time (less than five minutes) and a later refusal for questionnaire). In the end, 296 questionnaires were retained and used for analysis. Analysis of Variance (ANOVA) tests found no non-response bias or missing-value bias. Table 1 summarizes the sample characteristics. The samples are not over concentrated, which lays the foundation for our follow-up study.

**Table 1:** Characteristics of the research samples.

Size (Number of Employees)		Year of Establishment		Industry	
1–5 people	5.1%	1 year or less	8.8%	Agriculture	12.8%
6–20 people	19.2%	2–3 years	30.4%	Industry	49.0%
21–50 people	34.8%	4–5 years	36.8%	Service	38.2%
51–100 people	24.3%	6–8 years	24%		
>300 people	16.6%				

### Results of validity and reliability examination

SPSS 21.0 software was used to test the reliability of this study. The result indicates that Cronbach’s α of various dimensions is higher than the standard value 0.7 (see Tables 2–4), which shows that the variables of this study are highly internally consistent.

**Table 2:** Results of the reliability and convergent validity test of ethical structures.

Variables	Items	Factor loading coefficient	Cumulative variance explained rate	KMO	Bartlett's test	Cronbach’s α coefficient	Deleted Cronbach’s α coefficient	CR	AVE
Implicit ethical structure	Item (1)	0.799	61.183	0.848	0.000	0.842	0.801	0.890	0.612
	Item (2)	0.737					0.816		
	Item (3)	0.781					0.809		
	Item (4)	0.820					0.807		
	Item (5)	0.773					0.814		
Explicit ethical structure	Item (6)	0.843	75.077	0.838	0.000	0.888	0.877	0.923	0.750
	Item (7)	0.852					0.859		
	Item (8)	0.892					0.838		
	Item (9)	0.877					0.850		

**Table 3:** Results of the reliability and convergent validity test of entrepreneurial performance.

Variables	Items	Factor loading coefficient	Cumulative variance explained rate	KMO	Bartlett's test	Cronbach’s α coefficient	Deleted Cronbach’s α coefficient	CR	AVE
Survival	Item (1)	0.869	77.020	0.843	0.000	0.900	0.875	0.930	0.770
	Item (2)	0.858					0.876		
	Item (3)	0.878					0.877		
	Item (4)	0.904					0.856		
Sustainable growth	Item (5)	0.854	72.251	0.820	0.000	0.872	0.832	0.9123	0.723
	Item (6)	0.874					0.833		
	Item (7)	0.856					0.828		
	Item (8)	0.816					0.850		

**Table 4:** Results of the reliability and convergent validity test of firm visibility.

Variables	Items	Factor loading coefficient	Cumulative variance explained rate	KMO	Bartlett's test	Deleted Cronbach’s α coefficient	CR	AVE
Firm visibility	Item (1)	0.861	74.625	0.890	0.000	0.893	0.9362	0.746
	Item (2)	0.860				0.895		
	Item (3)	0.891				0.885		
	Item (4)	0.873				0.892		
	Item (5)	0.832				0.908		

As for content validity, first of all, the research subject was based on the studies of domestic and foreign researchers; it was checked and revised by experts of related fields. Second, the items were revised through the pilot test; some items were adjusted to enhance the clarity, technicality, and soundness of the survey instrument. Therefore, it is safe to say that variables are highly valid in content.

In this paper, SPSS 21.0 software was used to test the convergent validity of each measurement variable. The convergent validity can be illustrated by composite reliability (hereinafter referred to as CR) and average variation extracted from later variables (hereinafter referred to as AVE). The results are shown in Tables 2–4. The CR value of each variable is greater than 0.7, and the AVE value is greater than 0.5, all of which meet the relevant standards. The load factors obtained in the factor analysis are all greater than 0.5, and the proportion of the cumulative variance explained is greater than 50%, successfully meeting the research requirements.

Then, to test discriminant validity, Amos 24.0 software was utilized for confirmatory factor analysis. Since this paper aims to verify the different effects of entrepreneurial ethics on the survival and sustainable growth of new ventures, we conducted confirmatory factor analysis with the survival of new ventures as the dependent variable and other variables, as well as one with the sustainable growth of new ventures as the dependent variable and other variables, respectively. The results show that the four-factor model fitted the data reasonably better than any of the alternatives (Tables 5 and 6). Thus, the distinctiveness of the four constructs in the study was supported.

**Table 5:** Results of confirmatory factor analysis of the survival of new ventures as the dependent variable.

Model	cχ^2^	Df	c*Δ*χ^2^	TLI	CFI	RMSEA
Four-factor model	172.360	129		0.983	0.985	0.034
Three-factor model a	1162.912	133	990.552 **	0.600	0.652	0.162
Three-factor model b	697.324	132	524.964 **	0.779	0.809	0.120
Three-factor model c	862.704	132	690.344 **	0.714	0.753	0.137
Two-factor model d	1310.331	134	1137.971 **	0.546	0.602	0.173
Two factor model e	1377.383	134	1205.023 **	0.520	0.580	0.177
Two factor model f	1577.628	134	1405.268 **	0.443	0.512	0.191
One-factor model	2095.379	135	1923.019 **	0.249	0.337	0.222

** p < 0.01.

a: implicit ethical structure and firm visibility combined.

b: implicit ethical structure and survival combined.

c: implicit ethical structure and explicit ethical structure combined.

d: implicit ethical structure and firm visibility combined.

e: implicit ethical structure and survival combined.

f: implicit ethical structure and explicit ethical structure combined.

TLI, Tucker–Lewis Index; CFI, comparative fit index; RMSEA, root mean square error of approximation

**Table 6:** Results of confirmatory factor analysis of the sustainable growth of new ventures as the dependent variable.

Model	cχ^2^	Df	c*Δ*χ^2^	TLI	CFI	RMSEA
Four-factor model	180.726	129		0.978	0.982	0.037
Three-factor model a	1170.259	133	989.533**	0.576	0.631	0.163
Three-factor model b	744.477	132	563.751**	0.748	0.782	0.125
Three-factor model c	864.852	132	684.126**	0.698	0.739	0.137
Two-factor model d	1253.918	134	1073.192**	0.545	0.602	0.168
Two factor model e	1404.656	134	1223.93**	0.484	0.548	0.179
Two factor model f	1442.646	134	1261.92**	0.469	0.535	0.182
One-factor model	1962.825	135	1782.099**	0.263	0.350	0.214

** p < 0.01.

a: implicit ethical structure and firm visibility combined.

b: implicit ethical structure and sustainable growth combined.

c: implicit ethical structure and explicit ethical structure combined.

d: implicit ethical structure and firm visibility combined.

e: implicit ethical structure and sustainable growth combined.

f: implicit ethical structure and explicit ethical structure combined.

TLI, Tucker–Lewis Index; CFI, comparative fit index; RMSEA, root mean square error of approximation

### Common method bias

Harman’s single-factor method was applied to detect common method bias. The first extracted factor explains only 17.192% of the variance, which is less than the 50% common standard [81]. A single factor does not appear, and the dependent and independent variables are loaded on different factors, suggesting there is no single factor to explain multiple variances. Therefore, common method bias is unlikely to be a major threat here.

## Empirical results

### Results of the general descriptive analysis

Table 7 shows the descriptive statistics for each variable and the correlation coefficient matrix. As shown in the table, the average value of the variables is between 2 and 4 and has a low standard deviation, so that the variation range of each variable is small. Correlation coefficients between variables are all less than 0.7 [82], indicating that there is no significant multicollinearity among variables. The results of the correlation analysis are in line with the universal standards.

**Table 7:** Results of descriptive statistics and correlation coefficients.

Variables	1	2	3	4	5	6	7	8
1 Size								
2 Year	0.284**							
3 Industry	0.133*	0.072						
4 Implicit ethical structure	0.068	-0.065	0.009	(0.782)				
5 Explicit ethical structure	0.022	0.007	-0.189**	-0.050	(0.866)			
6 Firm visibility	-0.037	-0.036	0.053	0.002	0.091	(0.864)		
7 Survival	-0.003	-0.010	0.065	-0.063	-0.267**	-0.111	(0.877)	
8 Sustainable growth	0.099	0.061	0.005	0.150**	0.202**	-0.027	0.036	(0.850)
Mean value	3.280	2.760	2.250	3.926	3.752	3.849	3.342	2.771
Standard deviation	1.107	0.917	0.669	0.805	0.816	0.825	0.871	0.979

Note

* Correlation is significant at the 0.05 level (two-tailed).

** Correlation is significant at the 0.01 level (two-tailed). The diagonal is the square root of AVE.

### Multivariate linear regression models and results

In this paper, SPSS 21.0 was used to test the hypothesis by multivariate linear regression analysis. The threat of multicollinearity to the accuracy of the results needs to be eliminated before the regression analysis. The results of the analysis show that the maximum variance inflation factor (VIF) of the coefficient was 1.169, which was far lower than the critical value 10, and the tolerance value of each variable was greater than 0.5, reducing the threat of multicollinearity on the research results. Therefore, the data in this paper are suitable for multivariate linear regression analysis.

This paper studied the varying impacts of entrepreneurial ethics on the survival and sustainable growth of new ventures respectively. The regression results of entrepreneurial ethics on the survival of new ventures are shown in Table 8. The independent variables in Model 1 contained only control variables, and the dependent variable was the survival of new ventures. Model 2 added the variables of implicit ethical structure, explicit ethical structure, and firm visibility. The results of the empirical analysis showed that implicit ethical structure had no significant impact on the survival of new ventures (β = -0.078, p > 0.05) and the explicit ethical structure had a negative effect on the survival of new ventures (β = -0.258, p < 0.001), indicating that Hypothesis 1 is unsupported, while Hypothesis 2 is supported. In Model 3, entrepreneurial ethics are added, including the interaction term of implicit ethical structure and explicit ethical structure. The results showed that entrepreneurial ethics have a negative impact on the survival of new ventures (β = -0.253, p < 0.001), suggesting that Hypothesis 3 is supported.

**Table 8:** The logistic regression results of entrepreneurial ethics on survival.

Variables	Dependent variable: the survival of new ventures			
Model	Model 1	Model 2	Model 3	Model 4
Constant	-0.140	-0.029	-0.044	0.010
Control variables				
Size	-0.008	0.008	0.025	0.024
Year	-0.013	-0.021	-0.028	-0.035
Industry	0.067	0.022	0.014	0.001
Main research variable				
Implicit		-0.078	-0.103	-0.142**
Explicit		-0.258***	-0.193**	-0.234***
Firm visibility		-0.089	-0.087	-0.072
Implicit×Explicit			-0.253***	-0.262***
Implicit×Explicit×Firm Visibility				0.214***
Adjusted *R*^*2*^	-0.006	0.066	0.123	0.164
*ΔR*^*2*^	0.004	0.081	0.059	0.043
F change	0.435	8.513***	19.685***	15.013***

Note

** Correlation is significant at the 0.01 level (two-tailed).

*** Correlation is significant at the 0.001 level.

To test the moderation effect, we conducted a regression analysis using the procedure described by Baron and Aiken [83]. We have constructed the interaction term between entrepreneurial ethics and firm visibility; that is, implicit ethical structure × explicit ethical structure × firm visibility. Based on the previous analysis, Model 4 included the interaction terms (implicit × explicit × firm visibility) as the test for moderation. The results showed that firm visibility intensified the negative impact of entrepreneurial ethics on the survival of new ventures (β = 0.214, p < 0.001), indicating that Hypothesis 7 is supported.

The same method was used to study the impact of entrepreneurial ethics on the sustainable growth of new ventures. The results are shown in Table 9. In Model 6, the results of the empirical analysis showed that both implicit and explicit ethical structures had positive impacts on the sustainable growth of new ventures (β = 0.159, p < 0.01, β = 0.219, p < 0.001), indicating that both Hypotheses 4 and 5 are supported. In Model 7, the results of the empirical analysis showed that entrepreneurial ethics (the interaction of implicit and explicit ethical structures) had a positive impact on the sustainable growth of new ventures (β = 0.137, p < 0.05), hence Hypothesis 6 is supported.

**Table 9:** Logistic regression results of entrepreneurial ethics on sustainable growth.

Variables	Dependent Variable: the sustainable growth of new ventures			
Model	Model 5	Model 6	Model 7	Model 8
Constant	-0.337	-0.442	-0.432	-0.392
Control variables				
Size	0.090	0.064	0.054	0.053
Year	0.036	0.048	0.052	0.047
Industry	-0.010	0.035	0.039	0.031
Main research variable				
Implicit		0.159**	0.172**	0.146*
Explicit		0.219***	0.183**	0.156**
Firm visibility		-0.045	-0.045	-0.035
Implicit×Explicit			0.137*	0.131*
Implicit×Explicit×Firm Visibility				0.143*
Adjusted *R*^*2*^	0.001	0.060	0.074	0.090
*ΔR*^*2*^	0.011	0.068	0.017	0.019
F change	1.096	7.069***	5.491*	6.127*

Note

* Correlation is significant at the 0.05 level (two-tailed).

** Correlation is significant at the 0.01 level (two-tailed).

*** Correlation is significant at the 0.001 level.

By applying the same method as before to test the moderation effect, the results showed that in Model 8 that firm visibility had a positive impact on entrepreneurial ethics (the interaction of implicit and explicit ethical structures) for the sustainable growth of new ventures (β = 0.143, p < 0.05), indicating that Hypothesis 8 is supported.

**Fig 3:**
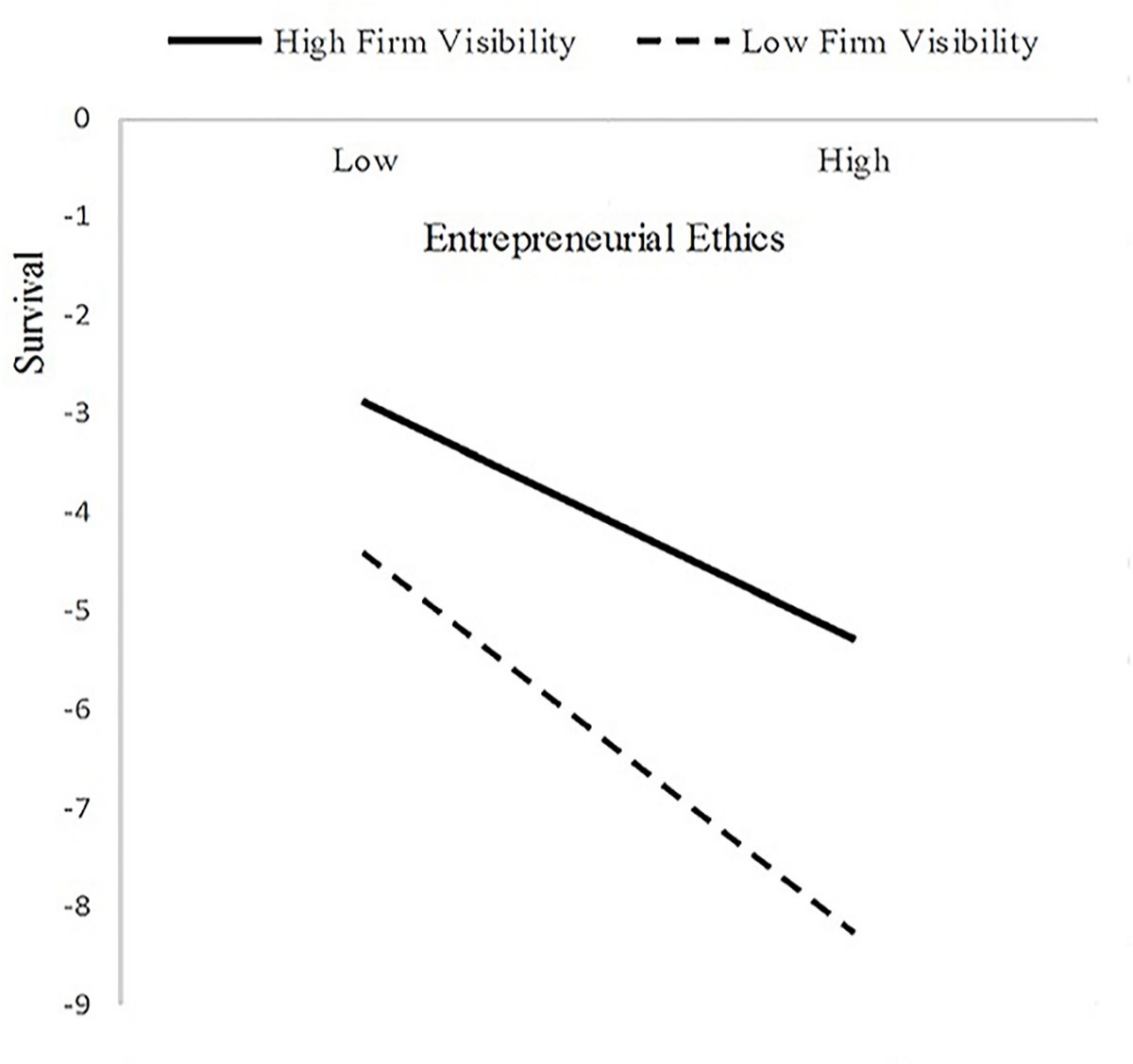
The moderating effects of firm visibility on the relationships between entrepreneurial ethics and the survival of new ventures.

**Fig 4:**
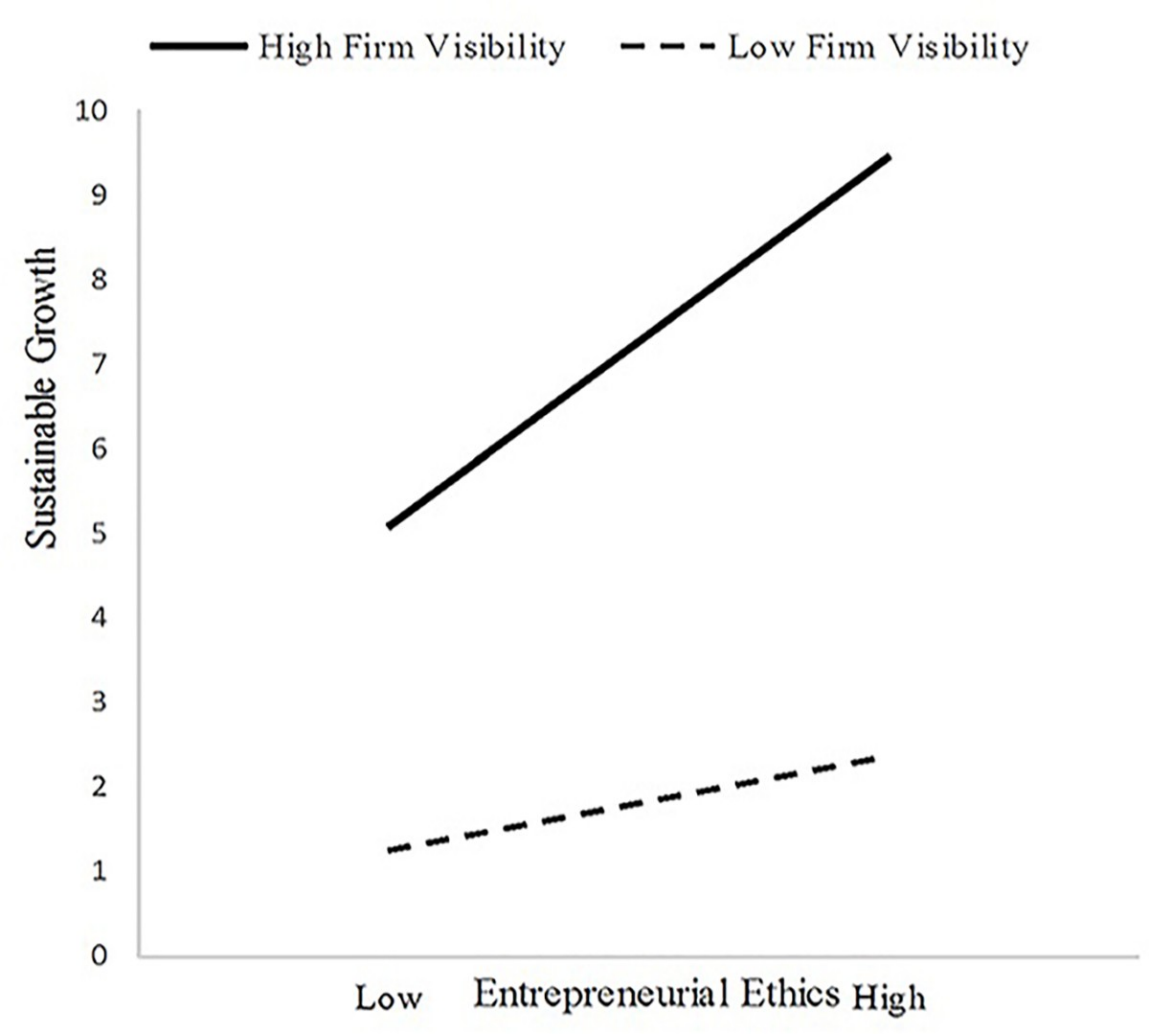
The moderating effects of firm visibility on the relationships between entrepreneurial ethics and the sustainable growth of new ventures.

To determine whether the forms of the interactions matched with those suggested by Hypotheses 7 and 8, in this paper, a simple slope analysis method proposed by Aiken [83] is used. This paper divided firm visibility into high firm visibility and low firm visibility, and then analyzed the effect of entrepreneurial ethics (implicit × explicit) on the survival and sustainable growth of new ventures under two different levels of firm visibility by regression analysis. In support of Hypothesis 7, we found that entrepreneurial ethics are more negatively related to the survival of new ventures when the firm had a lower level of firm visibility (simple slope test: β = -0.353, p <0.001) than when it has a higher level of firm visibility (simple slope test: β = -0.197, p <0.05) (Fig 3). Furthermore, in support of Hypothesis 8, entrepreneurial ethics were more positively related to the sustainable growth of new ventures when the firm had a higher level of firm visibility (simple slope test: β = 0.340, p <0.001) than when it had a lower level of firm visibility (simple slope test: β = 0.086, n.s) (Fig 4).

A summary of the results for all the hypotheses is shown in Table 10.

**Table 10:** Summary of the results for all the hypotheses.

Hypotheses	Results
H1: Implicit ethical structure has a negative impact on the survival of new ventures.	Not supported
H2: Explicit ethical structure has a negative impact on the survival of new ventures.	Supported
H3: Entrepreneurial ethics have a negative impact on the survival of new ventures.	Supported
H4: Implicit ethical structure has a positive impact on the sustainable growth of new ventures.	Supported
H5: Explicit ethical structure has a positive impact on the sustainable growth of new ventures.	Supported
H6: Entrepreneurial ethics have a positive impact on the sustainable growth of new ventures.	Supported
H7: Firm visibility can weaken the negative impact of entrepreneurial ethics on the survival of new ventures.	Supported
H8: Firm visibility can strengthen the positive impact of entrepreneurial ethics on the sustainable growth of new ventures.	Supported

## Discussion

This paper studied the different impact of the two structures–implicit ethical structure and explicit ethical structure–and their interaction on influencing entrepreneurial performance (survival and sustainable growth). Also, this paper investigated the regulating effect of firm visibility, which is of theoretical and practical significance to businesses.

### Theoretical contributions

This research made the following three theoretical contributions to business ethics studies. Firstly, this paper measures entrepreneurial ethics from the perspective of the internal ethical structure of new ventures, instead of ethical practice in the measurement of the previous research [5–6]. Such measurement can better reflect the essence of business ethics, being a concept derived from the internal ethical structure [12–13]. Furthermore, this study enriches the connotation of entrepreneurial ethics. In measurement of ethics, this paper finds that there are two kinds of internal ethical structures in new ventures: implicit ethical structure and explicit ethical structure. Compared with the implicit ethical structure, the explicit ethical structure of new ventures is less perfect, because the establishment of explicit ethical structure often needs to pay costs. Moreover, new ventures are faced with huge survival pressure [20]; explicit ethical structure is “expensive” for new ventures. Thus this finding confirms the lack of formal rules and regulations in the new ventures [13–14]. This paper also finds that the two ethical structures influence each other and complement each other. Their interaction constitutes the ethical values of new ventures, which is the embodiment of entrepreneurial ethics at the organizational level. Therefore, this discovery confirms the view that ethical concept derives from internal structure [12–13]. Different from the previous studies, which examined the role of the two ethical structures separately, this paper has a more systematic understanding of the enterprise’s internal ethical structures and their interaction.

Secondly, this paper divides entrepreneurial performance into two dimensions: survival and sustainable growth; and the paper focuses on the performance of new ventures. Thus, it makes up for the lack of dynamic investigation on the relationship between entrepreneurial ethics and entrepreneurial performance in previous studies. We find that entrepreneurial ethics is adverse to the survival of new ventures. Newness may restrict new ventures’ abilities to gain benefits from stakeholder relationships and positive reputations arising from ethical behavior. The establishment of ethical structures increases the operating costs of new ventures. We also find that entrepreneurial ethics can improve the sustainable growth of new ventures. As time passes by, ethical structures would contribute to an excellent internal ethical climate, thus increasing employees’ organizational commitment and improving their ability to innovate their performance. The above findings confirm the previous research conclusion that business ethics can promote the sustainable growth of enterprises [1]. Different from previous studies, this conclusion reflects the dynamic influence of ethics on new ventures, breaking through the previous single cognition of “good”or “bad” of entrepreneurial ethics and providing new ideas for explaining the theoretical differences of entrepreneurial ethics.

Thirdly, this paper puts firm visibility, which is the premise variable recognized by the institutional theory, into the research framework. In addition, this paper studies its moderating role in the relationship between entrepreneurial ethics and entrepreneurial performance, filling the research gap of institutional perspective on the relationship between ethics and performance. We find that high firm visibility is an important factor to amplify the positive effect of ethics. Empirical results showed that high firm visibility reduces the negative impact of entrepreneurial ethics on the survival of new ventures, and magnifies the positive impact of entrepreneurial ethics on the sustainable growth of new ventures. In contrast, the low firm visibility of new ventures is an important obstacle in the path of entrepreneurial ethics affecting entrepreneurial performance. Through simple slope analysis, we find that when the visibility of enterprises is low, the positive influence of entrepreneurial ethics on the sustainable growth of new ventures disappears. Because of the low visibility of new ventures, the ethical behavior of enterprises is not perceived by external stakeholders, and cannot obtain legitimacy, which greatly weakens the positive effect of ethics. The above findings confirm the positive moderating effect of good institutional environment on the relationship between CSR practice and performance [10–11]. Different from the previous studies, this paper has increased the diversity of cognition on the relationship between entrepreneurial ethics and entrepreneurial performance from the perspective of institution. Furthermore, it explored one specific situational factor, firm visibility that makes entrepreneurial ethics play a conducive role. This paper provides new insights into the relationship between internal ethics management and external institutional environment coordination.

### Managerial implications

The results of this study have important implications for the ethical management of new ventures and the formulation of government policies. First of all, this study finds that establishing a perfect ethical structure in the initial stage of the enterprise would generate huge survival pressure. However, in the long run, a proper ethical structure can help enterprises standardize the ethical behavior of employees and entrepreneurs, establishing a good image of an ethical company and forming a sustainable competitive advantage. Therefore, new ventures could establish a proper ethical structure based on the strategic positioning of long-term development. However, as explicit ethical structure needs relatively high costs, we suggest that new ventures should establish standardized explicit ethical structures according to their own business conditions. Such explicit ethical structure could involve making ethical guidelines to regulate employees’ behavior, writing ethical declarations to increase the recognition of customers and partners, and implementing regular ethical training. Consequently, the moral consciousness of employees and entrepreneurs would be improved continuously; the standard of moral for the enterprises would be formed finally. On the other hand, new ventures should not ignore the construction of implicit ethical structure. Entrepreneurs and senior managers should set moral examples, increase ethical dialogue among internal employees, and form a good moral climate. Additionally, we believe that the two ethical structures complement and promote each other. Therefore, new ventures should increase the understanding of the two structures and their interaction, form a closed-loop of mutual promotion between them, and maximize the synergy of the two ethical structures, thus promoting the sustainable performance of enterprises.

Secondly, based on the institutional theory, this study finds that firm visibility can weaken the negative impact of entrepreneurial ethics on the survival of new ventures and strengthen the positive impact of entrepreneurial ethics on the sustainable growth of ventures. Therefore, the government and society could improve the firm visibility of new ventures, help them to be recognized by more stakeholders, thus forming a virtuous circle that helps new ventures to be more ethical. For media and industry regulators, they should increase their focus on ethical behaviors of new ventures, reward ethical companies, and punish unscrupulous companies. For the government, it is necessary to strengthen the establishment of relevant laws and regulations, support ethical new ventures, and expand the influence of law. Through the above methods, the regulatory agencies and the ethical new ventures can gain more benefits. Inspiringly, a good social atmosphere can also be formed, benefiting the whole society. For enterprises, they can increase firm visibility by increasing advertising expenditure and increasing interaction with stakeholders. Therefore, the image of the ethical company could be known to more stakeholders and magnify the effective role of ethical management.

## Conclusion

It is still unclear whether new ventures’ performance benefits from entrepreneurial ethics and how they relate with each other. This paper explores the relationship between entrepreneurial ethics, firm visibility and entrepreneurial performance of new ventures from the institutional perspective. The impact of entrepreneurial ethics on entrepreneurial performance is explored in a dynamic way. Through the questionnaire survey method, we have obtained 296 effective questionnaires. The results show that: firstly, entrepreneurial ethics have a “two-sided” impact on entrepreneurial performance, which is unconducive to the survival of new ventures, but conducive to their sustainable growth. Secondly, firm visibility, as an institutional factor, plays a positive moderating role in the impact of entrepreneurial ethics on entrepreneurial performance. This conclusion confirms the usefulness of a good institutional environment and explores more beneficial factors to help ethics play an effective role in improving the entrepreneurial performance, so as to provide suggestions to guide the implementation of ethical management of the institution.

Although our study has explored the different effects of entrepreneurial ethics on the survival and sustainable growth of new ventures and has taken institutional factors into consideration, there are still some limitations. First of all, we used China for our data selection–a single country. Scholars have confirmed that enterprise ethics and CSR have a varying impact on performance in a different institutional environment. Therefore, the conclusions obtained in this paper cannot be fully representative. In the future, the scope of the data collection could be expanded to further test the hypotheses put forward in this paper. Secondly, firm visibility has many antecedents, such as enterprise advertising investment, media exposure, the degree of market development, etc. This paper measured “firm visibility” based only on a stakeholder perspective. Since firm visibility is affected by multiple variables, such as advertising intensity and market development, future research could measure it from multiple dimensions.

## Supporting information

S1 File Questionnaire information. (DOCX)

S1 Dataset Research data. (XLSX)
