# Supplementary material for: Does ethics really matter to the sustainability of new ventures? The relationship between entrepreneurial ethics, firm visibility and entrepreneurial performance
Source: PLoS One. 2020 Jan 28;15(1):e0226920. doi: 10.1371/journal.pone.0226920 (PMC6986731; doi:10.1371/journal.pone.0226920)
Supplement: S1 File — (DOCX) [file pone.0226920.s001.docx]

**Questionnaire information**

| **Personal and business information** | **Option** | | | | | |
| --- | --- | --- | --- | --- | --- | --- |
| Gender | Male | Female |  |  | |  |
| Education | High school and below | Specialty | Undergraduate | | Postgraduate and above |  |
| Position | Basic management | Middle management | Senior management | |  |  |
| company size (employee number) | 1-5 | 6-20 | 21-50 | | 51-100 | 100 |
| Company history (year) | Below 1 | 2-3 | 4-5 | | 6-8 | More than 8 |
| Company industry | Agriculture | Industry | Service industry | |  |  |

| **Questions** | **Strongly Disagree** | **Disagree** | **Undecided** | **Agree** | **Strongly Agree** |
| --- | --- | --- | --- | --- | --- |
| **Entrepreneurial ethics** |  |  |  |  |  |
| **Implicit ethical structure** |  |  |  |  |  |
| In our firm, the top manager is concerned about ethic. | 1 | 2 | 3 | 4 | 5 |
| Candid ethical dialogue takes place in our firm between management and employees. | 1 | 2 | 3 | 4 | 5 |
| In our firm, ethics is a topic of conversation among employee. | 1 | 2 | 3 | 4 | 5 |
| There are formal rewards for exemplary ethical behavior in our firm. | 1 | 2 | 3 | 4 | 5 |
| Stories concerning ethical employees are broadcasting throughout our firm. | 1 | 2 | 3 | 4 | 5 |
| **Explicit ethical structure** |  |  |  |  |  |
| There are ethical mission statements in our firm. | 1 | 2 | 3 | 4 | 5 |
| There are ethical codes of conduct in our firm. | 1 | 2 | 3 | 4 | 5 |
| There is a manager responsible for ethical issues in our firm. | 1 | 2 | 3 | 4 | 5 |
| There are training programs on ethical behavior in our firm. | 1 | 2 | 3 | 4 | 5 |
| **Entrepreneurial performance** |  |  |  |  |  |
| **The survival of new ventures** | 1 | 2 | 3 | 4 | 5 |
| Companies can cope with industry crises. | 1 | 2 | 3 | 4 | 5 |
| Companies can withstand cyclical changes to the economic environment. | 1 | 2 | 3 | 4 | 5 |
| A company’s market segments can respond to changes in industry development. |  |  |  |  |  |
| Companies can quickly recover from the shock of market changes to previous levels. | 1 | 2 | 3 | 4 | 5 |
| **The sustainable growth of new ventures** |  |  |  |  |  |
| The market share of the company’s products is growing rapidly. | 1 | 2 | 3 | 4 | 5 |
| The company's sales are growing rapidly. | 1 | 2 | 3 | 4 | 5 |
| The company’s profits are growing rapidly. | 1 | 2 | 3 | 4 | 5 |
| The number of employees is growing rapidly. | 1 | 2 | 3 | 4 | 5 |
| **Firm visibility** |  |  |  |  |  |
| Investors and shareholders pay close attention to our firm. | 1 | 2 | 3 | 4 | 5 |
| Labor Unions pay close attention to our firm. | 1 | 2 | 3 | 4 | 5 |
| External stakeholders such as consumers and suppliers pay close attention to our firm. | 1 | 2 | 3 | 4 | 5 |
| Regulatory agencies pay close attention to our firm. | 1 | 2 | 3 | 4 | 5 |
| External organizations such as the media and the community pay close attention to our firm. | 1 | 2 | 3 | 4 | 5 |

**问卷题项**

| 个人与企业信息 | 选项 | | | | | |
| --- | --- | --- | --- | --- | --- | --- |
| 性别 | 男性 | 女性 |  |  | |  |
| 学历 | 高中及以下 | 大专 | 本科 | | 研究生及以上 |  |
| 职位 | 基层管理人员 | 中层管理人员 | 高层管理人员 | |  |  |
| 公司规模（员工人数） | 1-5人 | 6-20人 | 21-50人 | | 51-100人 | 100人 |
| 公司年龄（年） | 低于1 | 2-3 | 4-5 | | 6-8 | 8人以上 |
| 公司行业 | 农业 | 工业 | 服务业 | |  |  |
|  |  |  |  |  |  |  |

| **问题** | **强烈反对** | **不同意** | **不确定** | **同意** | **强烈同意** |
| --- | --- | --- | --- | --- | --- |
| **创业伦理** |  |  |  |  |  |
| **内隐式伦理架构** |  |  |  |  |  |
| 公司的高层管理者重视伦理道德。 | 1 | 2 | 3 | 4 | 5 |
| 公司的管理层和员工会讨论伦理道德。 | 1 | 2 | 3 | 4 | 5 |
| 员工间会进行有关伦理道德的讨论。 | 1 | 2 | 3 | 4 | 5 |
| 公司会对道德模范进行实质的奖励。 | 1 | 2 | 3 | 4 | 5 |
| 公司内部流传着道德模范员工的事例。 | 1 | 2 | 3 | 4 | 5 |
| **外显式伦理架构** |  |  |  |  |  |
| 我们公司有明确的道德声明。 | 1 | 2 | 3 | 4 | 5 |
| 我们公司有道德行为准则。 | 1 | 2 | 3 | 4 | 5 |
| 公司有明确的负责道德审查的高层管理者。 | 1 | 2 | 3 | 4 | 5 |
| 公司有运营良好的针对商业道德的培训系统。 | 1 | 2 | 3 | 4 | 5 |
| **创业绩效** |  |  |  |  |  |
| **新创企业生存** | 1 | 2 | 3 | 4 | 5 |
| 公司可以应对行业危机。 | 1 | 2 | 3 | 4 | 5 |
| 公司能够应对外部经济环境的周期性变化。 | 1 | 2 | 3 | 4 | 5 |
| 公司的细分市场可以应对行业发展变化。 |  |  |  |  |  |
| 企业能快速从市场冲击中恢复到之前水平。 | 1 | 2 | 3 | 4 | 5 |
| **新创企业可持续成长** |  |  |  |  |  |
| 公司的市场份额增长迅速。 | 1 | 2 | 3 | 4 | 5 |
| 公司的销售额增长迅速。 | 1 | 2 | 3 | 4 | 5 |
| 公司的利润增长迅速。 | 1 | 2 | 3 | 4 | 5 |
| 公司的雇员人数增长迅速。 | 1 | 2 | 3 | 4 | 5 |
| **企业可见性** |  |  |  |  |  |
| 投资者和股东对我们公司密切关注。 | 1 | 2 | 3 | 4 | 5 |
| 工会对我们公司密切关注。 | 1 | 2 | 3 | 4 | 5 |
| 消费者和供应商等外部利益相关者对我们公司密切关注。 | 1 | 2 | 3 | 4 | 5 |
| 监管机构对我们公司密切关注。 | 1 | 2 | 3 | 4 | 5 |
| 媒体和社会等外部组织对我们公司密切关注。 | 1 | 2 | 3 | 4 | 5 |
